# Proteomic Charting of Imipenem Adaptive Responses in a Highly Carbapenem Resistant Clinical *Enterobacter roggenkampii* Isolate

**DOI:** 10.3390/antibiotics10050501

**Published:** 2021-04-28

**Authors:** Suruchi Nepal, Sandra Maaß, Stefano Grasso, Francis M. Cavallo, Jürgen Bartel, Dörte Becher, Erik Bathoorn, Jan Maarten van Dijl

**Affiliations:** 1 University Medical Center Groningen, Department of Medical Microbiology and Infection Prevention, University of Groningen, Hanzeplein 1, P.O. Box 30001, 9700 RB Groningen, The Netherlands; suruchinepal@gmail.com (S.N.); s.grasso@umcg.nl (S.G.); f.m.cavallo@rug.nl (F.M.C.); d.bathoorn@umcg.nl (E.B.); 2 Institute for Microbiology, University of Greifswald, Felix-Hausdorff-Str. 8, 17489 Greifswald, Germany; sandra.maass@uni-greifswald.de (S.M.); juergen.bartel@uni-greifswald.de (J.B.); dbecher@uni-greifswald.de (D.B.)

**Keywords:** carbapenem, *Enterobacter*, imipenem, resistance, SILAC, multi-omics

## Abstract

Gram-negative bacteria belonging to the *Enterobacter cloacae* complex are increasingly implicated in difficult-to-treat nosocomial infections, as exemplified by a recently characterized highly carbapenem-resistant clinical *Enterobacter roggenkampii* isolate with sequence type (ST) 232. While mechanisms of carbapenem resistance are well-understood, little is known about the responses of highly drug-resistant bacteria to these antibiotics. Our present study was therefore aimed at charting the responses of the *E. roggenkampii* ST232 isolate to the carbapenem imipenem, using a ‘stable isotope labeling of amino acids in cell culture’ approach for quantitative mass spectrometry. This unveiled diverse responses of *E. roggenkampii* ST232 to imipenem, especially altered levels of proteins for cell wall biogenesis, central carbon metabolism, respiration, iron–sulfur cluster synthesis, and metal homeostasis. These observations suggest a scenario where imipenem-challenged bacteria reduce metabolic activity to save resources otherwise used for cell wall biogenesis, and to limit formation of detrimental reactive oxygen species at the cytoplasmic membrane due to respiration and Fenton chemistry. We consider these observations important, because knowing the adaptive responses of a highly resistant bacterium of the *E. cloacae* complex to last-resort antibiotics, such as imipenem, provides a ‘sneak preview’ into the future development of antibiotic resistance in this emerging group of pathogens.

## 1. Introduction

Carbapenems are treasured as ‘last resort antibiotics’ that should only be used for treatment of infections when regular antibiotic regimens fail. Compared to other antibiotics, they display higher efficacies and a broader spectrum of activity against multidrug-resistant bacteria. The carbapenems belong to the class of β-lactam antibiotics for which penicillin binding proteins (PBPs) are the prime targets [1]. In Gram-negative bacteria, the PBP active sites reside in the periplasm between the inner and outer membranes, where they are of critical importance for the biosynthesis of the major cell wall component peptidoglycan [2]. Accordingly, PBP inhibition by β-lactam antibiotics leads to the weakening of the cell envelope and, ultimately, bacterial lysis [3,4]. In addition, it has been proposed that the activity of β-lactam antibiotics elicits oxidative stress, which may significantly contribute to bacterial killing [5,6,7].

Carbapenems, such as meropenem and imipenem, have the ability to remain stable in the presence of highly active bacterial β-lactamases and cephalosporinases. Unfortunately, the success of carbapenems in the fight against difficult-to-treat infections is now challenged by Gram-negative bacterial carbapenemases, which are the most effective known β-lactamases [8,9]. Enzymes belonging to this class hydrolyze the β-lactam ring, thereby producing an acidic derivative that lacks antibiotic activity. Three other mechanisms can add to the bacterial resistance against carbapenems. These include mutations that alter the expression and/or function of PBPs. Alternatively, the carbapenem concentration in the periplasm may be reduced by mutations impeding the function of porins that facilitate carbapenem passage through the bacterial outer membrane, or by overexpression of efflux pumps. Combinations of these mechanisms can lead to very high levels of carbapenem resistance in notorious pathogens of the family *Enterobacteriaceae*, such as *Escherichia coli* [10,11,12].

Recently, we have characterized a highly carbapenem-resistant clinical *Enterobacter roggenkampii* isolate with the sequence type (ST) 232, which belongs to the *E. cloacae* complex [13]. Our study showed that the carbapenem resistance of this isolate results from the production of an AmpC type cephalosporinase of the MIR family (i.e., MIR17) with carbapenemase activity, combined with truncation of the gene for an OmpC type porin [13]. We also showed that MIR17 was one of the most abundantly produced proteins detectable in our *E. roggenkampii* isolate.

While our previous study identified two key factors for carbapenem resistance in the *E. roggenkampii* ST232 isolate, it did not address the particular responses that this bacterium will show when challenged with a carbapenem. Therefore, in the present study, we asked the question whether, and if so, how this *E. roggenkampii* ST232 isolate would respond and adapt to a challenge with the carbapenem imipenem at a sub-inhibitory concentration. An answer to this question is important, because knowing the adaptive responses of a highly resistant pathogen to an antibiotic challenge may provide relevant clues for future therapeutic avenues. Moreover, the results may provide a ‘sneak preview’ into the future development of antibiotic resistance in bacteria belonging to the *E. cloacae* complex, a group of pathogens increasingly implicated in serious invasive nosocomial diseases [14]. Indeed, our experiments revealed several unexpected responses of our *E. roggenkampii* ST232 study isolate to imipenem, including reduced levels of PBPs, alterations in metal homeostasis, and several metabolic adaptations that can set a limit to the generation of reactive oxygen species (ROS).

## 2. Materials and Methods

### 2.1. Bacterial Isolate

The previously characterized *E. roggenkampii* ST232 isolate with strain number 339389L had been obtained from a rectal swab of a neonate [13]. Automated resistance analysis with the VITEK 2 system revealed increased MIC values to the carbapenems meropenem and imipenem. The whole-genome sequence of the 339389L isolate is available from NCBI with the accession number CP026536. Of note, the 339389L isolate was originally identified as *E. cloacae*, but closer inspection of its genome sequence for the present study revealed that, more precisely, it is an *E. roggenkampii* isolate that belongs to the *E. cloacae* complex.

### 2.2. Growth Media

Bacteria were grown on blood agar plates or in Roswell Park Memorial Institute 1640 medium (RPMI; Thermo Fisher Scientific, Waltham, MA, USA). RPMI medium deficient in both L-lysine and L-arginine was used for ‘stable isotope labeling of amino acids in cell culture’ (SILAC) experiments to perform quantitative mass spectrometry (MS). ‘Light’ (lysine 0, arginine 0) and ‘heavy’ amino acids (lysine 4 and arginine 6; Silantes, Munich, Germany) were, respectively, added to prepare the light and heavy RPMI media. The final concentration of lysine was 40 mg/L, while the final arginine concentration was 200 mg/L. As shown by MS, the incorporation rate of lysine 4 and arginine 6 was >90%.

### 2.3. SILAC and Protein Sample Preparation for Mass Spectrometry

**Figure 1:**
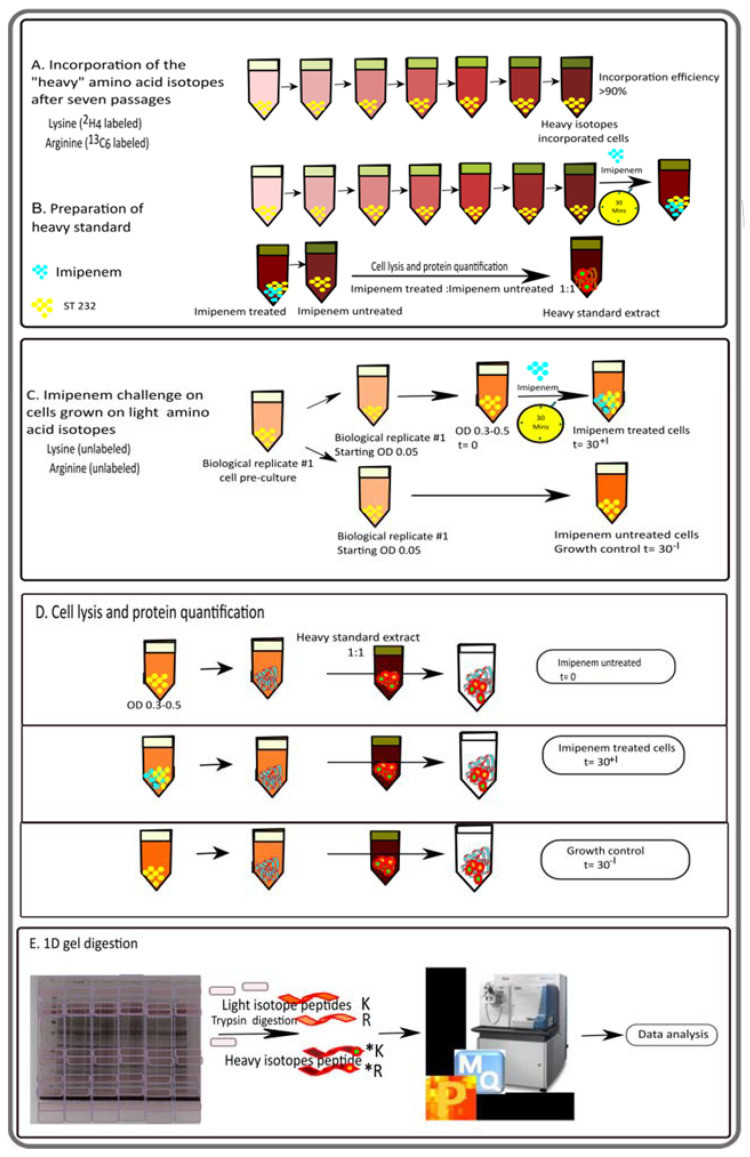
General overview of the experimental setup for the quantitative analysis of proteome changes in the *E. roggenkampii* ST232 study isolate in response to the presence of imipenem. (**A**) Schematic representation of the preparation of the heavy standard extract using a ‘stable isotope labeling of amino acids in cell culture’ (SILAC) LC-MS/MS approach. Bacteria were cultured in RPMI supplemented with heavy arginine and lysine for seven passages to achieve maximum incorporation (>90%). This ensured that all peptides containing heavy lysine and arginine were fully labelled. (**B**) On the seventh passage, the bacteria were treated with a sub-inhibitory amount of imipenem (5 mg/mL) for 30 min. Subsequently, the bacteria were collected, lysed, and the proteins were extracted. The final heavy standard was obtained by mixing the proteins from imipenem-treated and untreated cells, which facilitated the detection of the maximum number of proteins from both conditions. (**C**) For the actual imipenem-challenge experiment, the bacteria were grown in regular RPMI until an OD_600_ of 0.3–0.5, and samples were collected just before imipenem treatment (t = 0) and after 30 min of growth with (t = 30^+I^) or without imipenem (t = 30^−I^). (**D**) The collected cells were then lysed in the same manner as for the heavy standard extract, and proteins were collected. Lastly, the collected proteins were mixed with the heavy standard extract in a 1:1 ratio and separated by LDS-PAGE using 10% NuPAGE gels followed by MS analysis of the peptides eluted from the sliced gels (**E**). Three biological replicates were analyzed per sample group.

The experimental setup for SILAC experiments is schematically represented in Figure 1. To obtain a heavy protein standard, a bacterial pre-culture was grown at 37 °C under vigorous shaking (250 rpm) to an OD_600_ of 0.3 to 0.5. The cells were then grown for seven passages on heavy RPMI medium. The heavy-labeled cells were used to inoculate two cultures with heavy RPMI. From one of these cultures, the cells were harvested at an OD_600_ of 0.3 to 0.5 by centrifugation (~3000× *g*, 4 °C, 10 min) (Figure 1A). The other culture was used for a 30 min challenge with 5 mg/L imipenem (Sigma-Aldrich, St. Louis, MO, USA), after which, the cells were harvested by centrifugation (~3000× *g*, 4 °C, 10 min). Bacterial pellets were resuspended in bacterial protein extraction reagent (B-PER; Thermo Fisher Scientific, Waltham, MA, USA) according to the supplier’s instructions, and the mixture was agitated at 37 °C for 30 min. Cell debris was pelleted by centrifugation at 14,000× *g* for 20 min and the supernatant fraction with the extracted proteins was collected. The protein concentration was determined using the BCA protein assay kit (Thermo Fisher Scientific, Waltham, MA, USA). The heavy standard was then prepared by mixing proteins from imipenem-stressed and non-stressed cells in a 1:1 ratio based on protein amount (Figure 1B).

For the actual imipenem challenge experiments, bacteria were grown in light RPMI medium. To obtain three biological replicates for each condition, a pre-culture on light RPMI was used to inoculate six main cultures on light RPMI at a starting OD_600_ of 0.05 (Figure 1C). Growth was continued till an OD_600_ of 0.3 to 0.5. Subsequently, three cultures were challenged with 5 mg/L imipenem for 30 min, and samples were withdrawn prior to (t = 0) and after the addition of imipenem (t = 30^+I^). In parallel, the growth of the other three unchallenged cultures on light RPMI was continued to serve as a growth control (t = 30^−I^). Bacteria were collected from the culture samples by centrifugation (~3000× *g*, 4 °C, 10 min), and proteins were extracted with B-PER, as indicated above. The protein concentration was determined using the BCA protein assay kit.

Finally, 10 µg of the proteins extracted from cells grown on the light RPMI medium, with or without imipenem, were mixed with 10 µg of the heavy standard (Figure 1D). The protein mixtures thus obtained were separated by lithium dodecyl sulphate (LDS) polyacrylamide gel electrophoresis (PAGE) using 10% NuPAGE gels (Invitrogen, Carlsbad, CA, USA) (Figure 1E). Subsequently, the gels were stained with SimplyBlue SafeStain (Thermo Fisher Scientific, Waltham, MA, USA). Each LDS-PAGE lane was cut into 10 pieces, which were subsequently de-stained, desiccated, and rehydrated in trypsin as previously described [15]. Gel digests were incubated at 37 °C overnight. Peptides were eluted with water by sonication for 15 min and concentrated to 10 µL in a vacuum centrifuge.

### 2.4. Liquid Chromatography and Mass Spectrometry of SILAC Samples

Peptides were separated on an EASY-nLC II (Thermo Fisher Scientific, Waltham, MA, USA) system equipped with an in-house built 20 cm column (inner diameter 100 µm, outer diameter 360 µm) filled with ReproSil-Pur 120 C18-AQ reversed-phase material (3 µm particles, Dr. Maisch GmbH, Ammerbuch, Germany). The peptides were loaded with buffer A (0.1% acetic acid (*v*/*v*)) and subsequently eluted for 80 min using a 1% to 99% non-linear gradient with buffer B (0.1% acetic acid (*v*/*v*) in acetonitrile) at a flow rate of 300 nL/min. Eluted peptides were injected into a LTQ Orbitrap XL (Thermo Fisher Scientific, Waltham, MA, USA). A survey scan at a resolution of R = 30,000 was followed by selection of the five most abundant precursor ions for fragmentation. The mass spectrometry proteomics data have been deposited to the ProteomeXchange Consortium via the PRIDE partner repository [16] with the dataset identifier PXD013412.

### 2.5. SILAC Data Analysis

Relative protein quantification was achieved using the MaxQuant software (version 1.6.2.10.) [17] and the Andromeda plug-in [18]. The *.raw files were searched against a protein database that was based on the genome sequence of the *E. roggenkampii* ST232 isolate, including 5037 protein entries. Additionally, MaxQuant’s generic contamination list was included during the search. The database search was performed with the following parameters: digestion mode; trypsin/P with up to 2 missed cleavages; Arg +6 and Lys +4 specified as MS1 labels; variable modification; methionine oxidation; and maximal number of 5 modifications per peptide and activated ‘match-between-runs’ feature. The false discovery rates of peptide spectrum match and protein were set to 0.01. Only unique peptides were used for protein quantification. The identified proteins from MaxQuant output files were filtered for contaminants, identified only by site and reverse hits with the Perseus software (v. 1.6.1.3). Proteins were accepted if at least two unique peptides could be identified in at least two of the three biological replicates. The heavy-to-light ratios were log2-transformed, exported from MaxQuant, and used for statistical analysis using TM4 (Saeed et al., 2003). Statistical significance required a *p*-value < 0.01 in a student’s t-test. The identified proteins and their altered abundances are presented in Supplementary Table S1. Absolute protein quantification was performed using the iBAQ algorithm as described by Schwanhäusser et al. [19], and the results are presented in Supplementary Table S2.

### 2.6. In Silico Functional Proteome Annotation

Based on our previously published de novo hybrid genome assembly and RAST annotation [13], the predicted proteome of the *E. roggenkampii* ST232 isolate was functionally annotated in silico. First, InterPro Scan version 5.33 [20] was used against the InterPro consortium database version 72 [21] to detect all known protein domains. Subsequently, a search for all domains related to PBPs and/or β-lactamases was performed. Putative positive hits for proteins of the two families were also checked for the presence of orthologues in the type strain *E. cloacae* ATCC 13047. A reciprocal best hits analysis to detect homologues was performed as previously described [13]. The functional annotation of predicted proteins was verified by blastp analyses of the respective amino acidic sequences using the NCBI nr database (default parameters) [22] to identify known protein homologues from closely related species. To further functionally characterize the predicted proteome, ‘clusters of orthologous groups’ (COG) functional categories were predicted using eggNOG-mapper version 1 with the DIAMOND mapping mode and default settings [23] against the eggNOG database version 4.5.1 [24].

### 2.7. Pathway Analyses

*E. roggenkampii* ST232 proteins present at differential levels in the presence or absence of imipenem, as identified by MS, were allocated to particular pathways based on the Kyoto Encyclopedia of Genes and Genomes (KEGG; https://www.genome.jp/kegg/pathway.html; accessed on 13 June 2019) [25]. Subsequently, the pathways thus identified were complemented by MS-identified non-regulated proteins and proteins predicted by in silico proteome annotation.

### 2.8. Identification of Fur Boxes in the E. roggenkampii Genome Sequence

To identify potential binding sites for the Fur repressor (i.e., *fur* boxes), a position specific scoring matrix (PSSM) was used, which detects specific DNA sequence motifs. The applied PSSM was specific to *E. coli*, and had previously been successfully used on *Salmonella typhimurium* [26]. A scan search on both strands was carried out using the PoSSuMearch2.0 algorithm [27], assuming a uniform character distribution in the genome. In addition, the *LazyDistrib* algorithm and lookahead scoring were integrated into the search tool. All hits with an E-value > 100 were considered and manually curated based on their position in the genome, direction, and proximity to the annotated genes (Supplementary Table S1).

### 2.9. Inductively Coupled Plasma Mass Spectrometry (ICP-MS) Analyses

For ICP-MS analyses to determine changes in the bacterial metal content upon imipenem challenge, the *E. roggenkampii* ST232 isolate was first grown overnight at 37 °C on blood agar plates. Then, 150 mL RPMI medium was inoculated in triplicate with five colonies and the resulting cultures were grown at 37 °C, 250 rpm. After 4 h of growth (OD_600_ 0.07), the three cultures were pooled and an aliquot of 150 mL was collected for ICP-MS analysis (t = 0 sample). The remaining 300 mL was split into two separate cultures of 150 mL, one of which was supplemented with 5 mg/L imipenem, whereas the other received no antibiotic, and growth was continued for 30 min. Cells from these cultures were used to prepare the t = 30^+I^ and t = 30^−I^ samples, respectively. To comply with biosafety regulations, prior to further processing of the t = 0, t = 30^+I^, and t = 30^−I^ samples, the bacteria were killed by 30 min exposure to ultraviolet light. Bacteria were collected by centrifugation (~3000× *g*, 4 °C, 10 min), washed in 1 mL 10 mM Tris-HCl buffer (pH 8.0), and pelleted by centrifugation. This procedure was carried out three times to obtain biological replicates. Cell pellets were stored at −20 °C until mechanical disruption with a FastPrep-24 (MP Biomedicals, Irvine, CA, USA) in six cycles of 30 sec (6.5 m/s) in 10 mM Tris-HCl buffer (pH 7.5). Cell debris and glass beads were removed by two subsequent centrifugation steps (5000× *g*, 5 min and 20,000× *g*, 10 min, 4 °C). A 20 µL aliquot was then separated by gel filtration on a Superose 6 Increase 3.2 × 300 column (Sigma-Aldrich, St. Louis, MO, USA; eluent: homogenization buffer at 100 µL min^−1^) and the flow-through was directly injected into a 7500c ICP-MS (Agilent Technologies, Santa Clara, CA, USA) to monitor isotope intensities essentially as previously described [28]. The plasma was operated at 1420 W and other parameters were optimized as necessary. The chromatograms were further corrected for sensitivity drifts by a smoothed ^13^C baseline and for the natural isotope abundance, as provided by the instrument manufacturer, using R scripts. Peak fitting and integration were performed with the program Fityk (version 1.3.1) [29] and peak areas were normalized to the protein content of the sample as determined by Bradford assay. The results or the ICP-MS analyses are presented in Supplementary Table S3.

### 2.10. Biological and Chemical Safety

*E. roggenkampii* is a biosafety level 2 (BSL-2) microbiological agent, and was accordingly handled following appropriate safety procedures. All experiments involving live *E. roggenkampii* bacteria and chemical manipulations of *E. roggenkampii* protein extracts were performed under appropriate containment conditions and protective gloves were worn. All chemicals and reagents used in this study were handled according to the local guidelines for safe usage and protection of the environment.

### 2.11. Data Availability

The whole-genome sequence of the *E. roggenkampii* isolate 339389L is available from NCBI with the accession number CP026536. The mass spectrometry proteomics data have been deposited to the ProteomeXchange Consortium via the PRIDE partner repository [16] with the dataset identifier PXD013412.

## 3. Results and Discussion

### 3.1. Analysis of the Imipenem Stress Response of E. roggenkampii ST232 by Stable Isotope Labeling

To determine how the carbapenem-resistant *E. roggenkampii* ST232 study isolate responded to imipenem at a concentration just below the MIC of 8 mg/L, we established a SILAC approach, as schematically represented in Figure 1. To this end, we first prepared a so-called ‘heavy standard’ protein extract of *E. roggenkampii* grown with or without imipenem in RPMI medium in the presence of heavy isotope-labeled lysine and arginine. Next, we performed the actual experiment, where a culture with exponentially growing bacteria in regular RPMI medium was divided into two moieties, where one was challenged with a sub-inhibitory concentration of imipenem (5 mg/L) for 30 min whereas the other was not exposed to imipenem. Samples were collected prior to the addition of imipenem (t = 0) and after 30 min of imipenem exposure (t = 30^+I^), or upon continued growth in the absence of imipenem (t = 30^−I^). For reproducibility, the experiment was performed in triplicate, so that, for each sampling condition, three biological replicate samples were obtained, resulting in nine samples in total. Subsequently, these samples were spiked with the heavy protein extract and the protein content was analyzed by quantitative MS. Of note, we selected the cell culture medium RPMI for this experiment as previous studies have shown that, from a bacterial perspective, RPMI closely resembles the conditions in human plasma [30]. Hence, the growth on RPMI mimics the situation encountered by *E. roggenkampii* upon invasive growth. Furthermore, we opted for a relatively short imipenem exposure time of 30 min in order to capture the initial response of *E. roggenkampii* to the presence of imipenem, rather than late responses where many changes in protein composition may be related to secondary effects elicited by the primary responses. The 30-min imipenem exposure time was also relatively short compared to the doubling time of 56 min that *E. roggenkampii* displays upon culturing in RPMI, which minimizes the influence of growth-related effects or altered cell viability on the protein composition. Additionally, imipenem is fairly rapidly eliminated from the human body, with an estimated half-life of ~60 min in adults, which focuses attention on the bacterial responses within this time frame [31].

**Figure 2:**
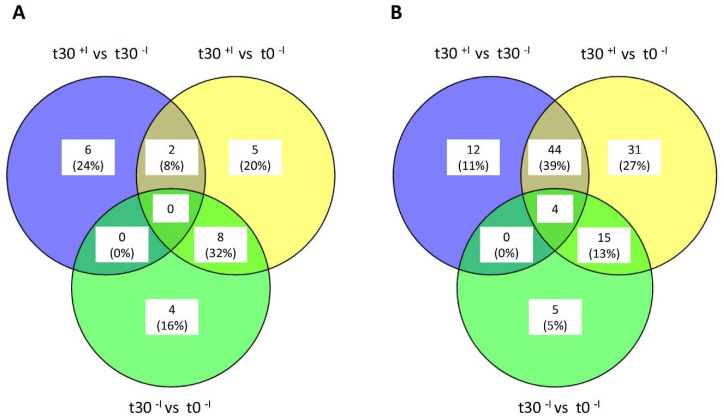
Venn diagrams of differentially regulated proteins of *E. roggenkampii* ST232 upon an imipenem challenge of 30 min. (**A**) Unique and shared upregulated cellular proteins in the three investigated conditions: (i) t = 30^+I^ vs. t = 30^−I^; (ii) t = 30^+I^ vs. t = 0; and (iii) t = 30^−I^ vs. t = 0. (**B**) Unique and shared downregulated cellular proteins in the three investigated conditions: (i) t = 30^+I^ vs. t = 30^−I^; (ii) t = 30^+I^ vs. t = 0; and (iii) t = 30^−I^ vs. t = 0.

In total, 864 *E. roggenkampii* proteins were identified by MS analysis of the SILAC samples (Supplementary Table S1). These included 28 ‘on–off’ proteins that were detectable in only one condition over all. Assessment of proteins with log2 fold changes higher than 0.8 showed that the abundance of relatively few proteins was significantly changed due to the presence of imipenem, as summarized in the Venn diagrams presented in Figure 2. Specifically, upon comparison of the t = 30^+I^ and the t = 30^−I^ samples, we observed that the levels of eight proteins were consistently increased, while the levels of 56 proteins were decreased (*p* < 0.01). To assess proteomic changes related to growth of *E. roggenkampii* over 30 min, the t = 0 and t = 30^−I^ samples were compared, showing increased levels of 12 proteins and decreased levels of 24 proteins. Furthermore, comparison of the t = 0 and t = 30^+I^ samples revealed increased levels of 15 proteins and decreased levels of 94 proteins as a consequence of growth over 30 min in the presence of imipenem. Importantly, the proteins for which different levels were evident upon comparison of the t = 0 and t = 30^+I^ samples include various proteins that present altered abundance due to 30 min growth. Therefore, we focused our further analysis of imipenem-specific responses in our *E. roggenkampii* ST232 study isolate on the regulated proteins identified by comparison of the t = 30^+I^ and t = 30^−I^ samples.

### 3.2. Biological Processes Adapted in Response to Imipenem Challenge

**Figure 3:**
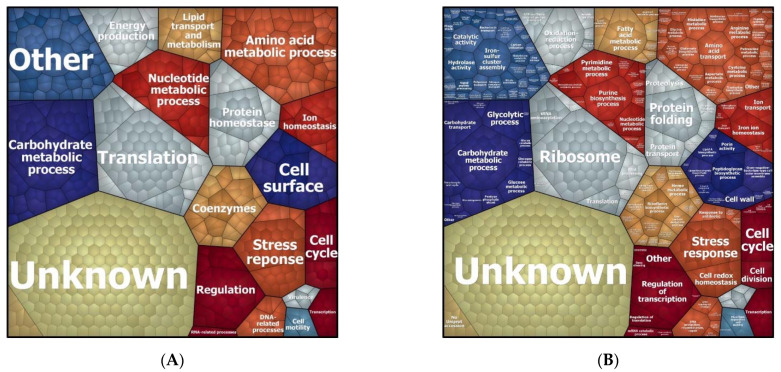
Voronoi treemaps presenting the functional categories of all proteins of *E. roggenkampii* ST232 as identified in our quantitative proteome analysis. (**A**) Treemap based on gene ontology, showing clusters of orthologous protein groups (COGs) at the general functional level 1. (**B**) Treemap at the functional level 2 showing the biological processes in which the identified proteins are involved as assigned by gene ontology (GO).

**Figure 4:**
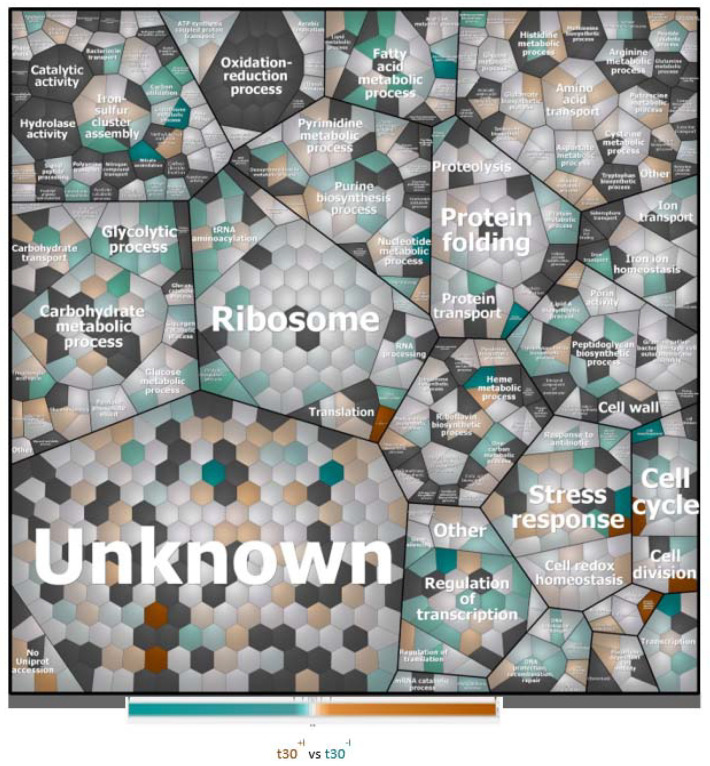
Voronoi treemaps presenting the functional categories of up-, down-, and non-regulated proteins in *E. roggenkampii* ST232 upon imipenem challenge. Treemap showing the up- and down-regulated proteins of imipenem-challenged bacteria marked in color code. For each protein, the relative amount was assessed based on the log2-transformed heavy-to-light ratios as exported from MaxQuant, and statistically analyzed using TM4.

To understand which biological processes are modulated by a sub-inhibitory concentration of imipenem, we first attributed all identified proteins (i.e., the regulated plus the non-regulated proteins) to biological processes according to gene ontology, and graphically grouped them into related clusters using Voronoi treemaps. This allows for their inspection at different functional levels, as represented in Figure 3 and Supplementary Figure S1. This approach showed that, apart from proteins of unknown function, the highest numbers of identified proteins are involved in translation and the metabolism of carbohydrates, amino acids, and nucleotides. Other well-represented functional protein categories relate to cell wall biogenesis, ion homeostasis, and cellular stress responses. Interestingly, the proteins displaying significantly changed levels in the presence of imipenem turned out to be associated with all of these functional categories, as shown in Figure 4.

### 3.3. The AmpC MIR17 Is Hyper-Expressed, but Not Inducible by Imipenem

Our previous investigations indicated that the AmpC cephalosporinase with carbapenemase activity MIR17 is abundantly produced in the *E. roggenkampii* ST232 study isolate [13]. Furthermore, it was observed that the resistance of this isolate to imipenem had to be largely attributed to the production of MIR17. This raised the question as to whether MIR17 might be regulated in response to the presence of imipenem. However, our quantitative proteome data did not show a significant regulation of MIR17 (Supplementary Table S1). To verify this finding, and to assess the relative abundance of MIR17 amongst the other proteins produced by the *E. roggenkampii* ST232 study isolate, we quantified bacterial proteins from samples of cells grown in light RPMI medium that were collected at t = 0, t = 30^+I^, and t = 30^−I^, based on iBAQ values. This showed that MIR17 is actually the most abundant protein detectable in the investigated *E. roggenkampii* isolate, and that its abundance did not vary in the presence of imipenem (Supplementary Figure S2 and Table S2). To explain this observation, we verified the integrity of genes known to regulate the expression of *ampC* genes in bacteria belonging to the *E. cloacae* complex. Indeed, we observed that an IS element encoding an IS26 family transposase had integrated into the *ampD* gene (Gene ID: 45794411) of our study isolate. AmpD is a cytoplasmic N-acetylmuramyl-l-alanine amidase that modulates the activity of the AmpR repressor of *ampC* gene expression, and *ampD* mutations were previously shown to lead to extremely high levels of *ampC* expression, even in the absence of β-lactams [32]. We therefore conclude that MIR17 is constitutively and highly expressed, at least under the applied experimental conditions.

### 3.4. Function of Proteins Differentially Expressed in the Presence of Imipenem

As indicated above, the levels of eight proteins in total were significantly increased when the *E. roggenkampii* ST232 study isolate was challenged with imipenem (Supplementary Table S1). The highest level of upregulation was observed for the FMN oxidoreductase NamA. Other significantly upregulated proteins, in order of their upregulation ratio from high to low, were the ribonucleotide reductase subunit NrdB, the lactoylglutathione lyase GloA, the copper resistance protein PcoC, the histidinol dehydrogenase HisD, the phosphopentomutase DeoB, the ferric uptake regulation protein Fur, and the 1,4-dihydroxy-2-naphthoyl-CoA hydrolase MenI. Interestingly, NamA and GloA are involved in cellular detoxification processes, while PcoC and Fur are involved in metal homeostasis.

Fifty-six proteins displayed significantly reduced levels in the presence of imipenem. These proteins are involved in cell wall, membrane, and cell envelope biogenesis (GalU, GlmU, MrcB, Prc); signal transduction mechanisms (ArcA, OmpR); carbohydrate transport and metabolism (Gnd, ManX, PfkA, Pgk, PtsI, PykA); energy production and conversion (AceF, AdhE, BetB, LpdA, MaeB, Pta, QorA); lipid transport and metabolism (AccD); amino acid transport and metabolism (AspA); coenzyme transport and metabolism (FolD, NadE); inorganic ion transport and metabolism (CynT, Dps, HmuS), nucleotide transport and metabolism (Amn, PurC), replication and repair (GyrA, ParC, Rob, SeqA); secondary metabolite and transport (SufC); transcription (Lrp, NusA, RpoB); translation, ribosomal structure, and biogenesis (AlaS, AsnS, InfB, LeuS, LysS, Pnp, Rph, RpsA, ThrS); posttranslational modification, protein turnover, and chaperoning (FtsH, HflC, HflK, HtpG, Lon, SufB, YibF); and intracellular protein trafficking, secretion, and vesicular transport (FtsY, TolQ). Four proteins present at reduced levels are categorized as unknown, but Blast searches suggest roles for these proteins in translation (Ent638_3316, EttA, StpA, YihX).

### 3.5. Potential Impact of Imipenem on Cell Wall Biogenesis

**Figure 5:**
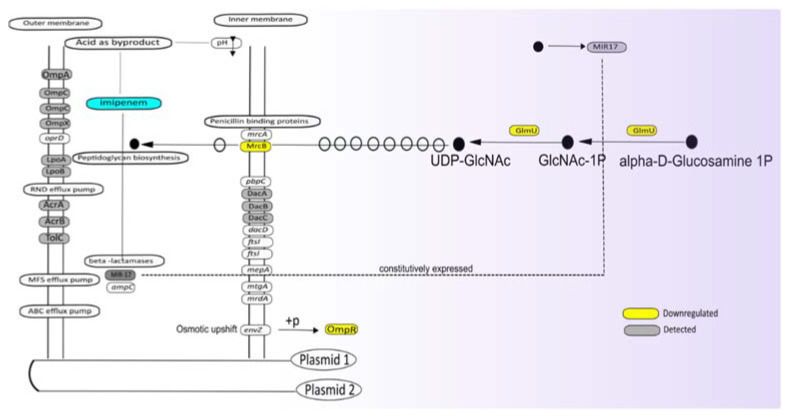
Graphical representation of proteins involved in cell envelope biogenesis and β-lactam resistance based on genomic and proteomic analyses. In those cases, where a particular gene was identified, but not the respective protein, the gene name is indicated in the respective box. If a protein was identified but not significantly regulated, the respective box is marked with gray shading, and if a protein was found to be present at an altered level at t = 30^+I^ compared to t = 30^−I^, the respective box is marked in color code. Yellow indicates down-regulation.

The assignment of proteins detected at altered levels in the presence of imipenem to functional categories provides important clues about the mechanisms employed by our *E. roggenkampii* ST232 study isolate for dealing with the detrimental effects of this antibiotic. In the first place, imipenem is a potent inhibitor of cell wall biogenesis, blocking either the transpeptidation or carboxylation reactions catalyzed by PBPs [33]. As a consequence, the peptidoglycan layer of the growing bacteria will be weakened, which may lead to cell death by lysis, especially in hypotonic environments [4]. In general, bacteria encode multiple PBPs [2], and this is also true for the investigated *E. roggenkampii* isolate. A detailed inspection of its genome sequence revealed multiple PBP-encoding genes on the chromosome, in particular, *mrcA* (PBP1A), *mrcB* (PBP1B), *pbpC* (PBP1C), *mrdA* (PBPB), *ftsI* (PBP3), *dacC*, and *dacD* for type 5 and type 6 low-molecular weight PBPs, and *mtgA* (Glycosyltransferase), as schematically represented in Figure 5 (Supplementary Table S4). Four of the respective PBPs, namely MrcB, DacA, DacB, and DacC, were identified in our proteome analyses (Figure 5). In this respect, it should be noted that the identification of membrane proteins by MS is technically challenging, especially in the case of low-abundant membrane proteins.

Interestingly, in the presence of imipenem, the cells showed a strong inhibition of MrcB, while the levels of DacABC were not influenced. MrcB catalyzes the transglycosylation and transpeptidation of peptidoglycan, necessary for the formation of the sacculus that is critical in the protection of the bacterial cell against osmotic lysis. Of note, the bifunctional GlmU protein involved in cell wall biogenesis was also significantly downregulated. GlmU is known to catalyze the last two sequential reactions in the de novo biosynthetic pathway for UDP-N-acetylglucosamine (in short UDP-GlcNAc), an essential precursor of lipid II. Together, these findings suggest that the bacteria reduce their capacity for cell wall biogenesis in response to the presence of imipenem. This could lead to some osmotic stress, which is sensed by the bacteria. Consistent with this idea, in the presence of imipenem, we observed a downregulation of the ‘outer membrane porin regulator’ OmpR. OmpR is the response regulator of the EnvZ–OmpR two-component regulatory system, which senses osmotic stress. In *E. coli*, EnvZ and OmpR are downregulated in response to low-osmolarity conditions [34]. Nevertheless, we did not observe the regulation of typical OmpR-controlled porins like OmpC (Supplementary Table S1).

Peptidoglycan biogenesis is a costly process in terms of cellular resources, and it has been proposed that blocking peptidoglycan synthesis will result in the rerouting of phosphorylated sugars from anabolism towards catabolism [5]. In turn, this would result in increased tricarboxylic acid (TCA) cycle activity and respiration, with excessive formation of ROS as a consequence. Indeed, several studies have shown that inhibition of cell wall biogenesis can lead to severe lipid damage by ROS from the respiratory chain [5,35,36]. This could actually be one of the main bactericidal effects of β-lactam antibiotics, since peptidoglycan, as well as its precursors and turnover products, may serve as scavengers of ROS [35]. In this respect, the presently observed downregulation of enzymes involved in the synthesis of peptidoglycan could potentially allow the bacteria to overcome a detrimental depletion of resources due to reduced PBP activity in the presence of imipenem.

### 3.6. Potential Impact of Imipenem on Central Carbon Metabolism and Respiration

**Figure 6:**
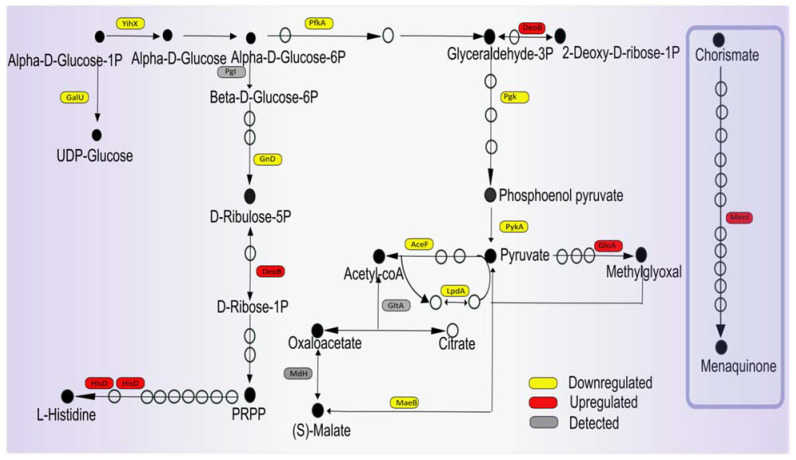
Graphical representation of proteins involved in central carbon metabolism with altered abundance upon imipenem challenge. The indicated proteins are active in the glucose metabolism, pyruvate dehydrogenase, citric acid cycle, or pentose phosphate pathway. Boxes marked in yellow represent downregulated proteins, red marks upregulated proteins, and gray marks detected proteins that show no regulation in the presence of imipenem. Filled bullets mark the main pathway products and open bullets mark the intermediate products.

Reduced catabolic activity would be an alternative way of limiting ROS production upon impaired peptidoglycan production, and our present observations imply that the bacteria indeed follow this strategy when confronted with imipenem. For example, ManX, a component of the phosphoenolpyruvate-dependent sugar phosphotransferase system, is downregulated, suggesting that sugar uptake may be limited. In addition, the imipenem-treated bacteria produce reduced levels of the α-D-glucose-1-phosphate phosphatase YihX, which catalyzes the dephosphorylation of α-D-glucose-1-phosphate (Glc1P) to α-d-glucose as a substrate for glycolysis [37,38]. Even more importantly, the levels of the ATP-dependent 6-phosphofructokinase PfkA, catalyzing the first committing step of glycolysis, are reduced, and the same applies to the ATP-generating glycolytic enzymes phosphoglycerate kinase Pgk and pyruvate kinase PykA (Figure 6). Consistent with the potentially reduced glycolysis, the lactoylglutathione lyase GloA is upregulated, which serves to prevent the accumulation of the toxic methylglyoxal by the methylglyoxal pathway upon reduced glycolytic activity (Figure 6).

The imipenem-treated bacteria also reduce the level of the NADP-dependent malic enzyme MaeB, which is responsible for the conversion of malate to pyruvate (Figure 6), suggesting that this reduces the influx of pyruvate into the TCA cycle. Related to this may be the observed decrease in the level of the response regulator AcrA, which is part of the ArcA–ArcB two-component regulatory system that responds to the respiratory/fermentative state of the cell by sensing the redox states of the quinone pools. Indeed, in *E. coli*, the *maeB* gene is repressed by ArcA–ArcB under fermentative conditions [39].

Together with a reduced availability of the pyruvate dehydrogenase complex component AceF in the presence of imipenem, the adaptations in the bacterial potential for glycolysis, as described above, will, most likely, lead to a reduction in the TCA cycle-mediated conversion of NAD^+^ to NADH. Accordingly, it seems to make sense that the bacteria downregulate production of the NAD^+^ synthase NadE, thereby setting a limit to the cellular levels of NADH that are available for aerobic respiration. In turn, this could reduce the unwanted generation of ROS. The aforementioned reduction in the level of MaeB may also serve to limit ROS production, as it has been reported that NADP-dependent malic enzymes play important roles in the neutralization of ROS upon increased metabolic activity [40,41]. Another interesting observation is the upregulation of the 1,4-dihydroxy-2-naphthoyl-CoA hydrolase MenI in the bacteria exposed to imipenem, which is involved in menaquinone (vitamin K) biosynthesis as part of the cellular quinol/quinone metabolism (Figure 6). Of note, vitamin K is a known antioxidant that suppresses lipid peroxidation [42]. Thus, the observed upregulation of MenI may help the bacteria to limit the detrimental effects of ROS produced by respiration.

In contrast to the catabolic pathways, certain anabolic pathways, e.g., for purine and pyrimidine synthesis, seem to be upregulated in the presence of imipenem. This is exemplified by increased levels of the phosphopentomutase DeoB, which catalyzes the phosphotransfer between the C1 and C5 carbon atoms of pentose, producing phosphoribosyl pyrophosphate (PRPP; Figure 6). In accordance with the fact that PRPP is needed for the synthesis of histidine, the HisD protein involved in the NAD^+^-dependent oxidations of L-histidinol to L-histidinaldehyde is also increased. Nonetheless, the imipenem-exposed bacteria displayed decreased levels of the 6-phosphogluconate dehydrogenase Gnd (Figure 6), which decarboxylates 6-phosphogluconate to ribulose 5-phosphate and CO_2_ and reduces NADP to NADPH. This suggests a lowering in the NADPH levels, which could be exacerbated by the strongly increased levels of the FMN oxidoreductase NamA, which catalyzes the conversion of NADPH to NADP^+^. However, NamA has also been implicated in cellular detoxification processes, so the increased NamA level may reflect an as yet undefined NADPH-dependent detoxification process needed to respond to the presence of imipenem.

**Figure 7:**
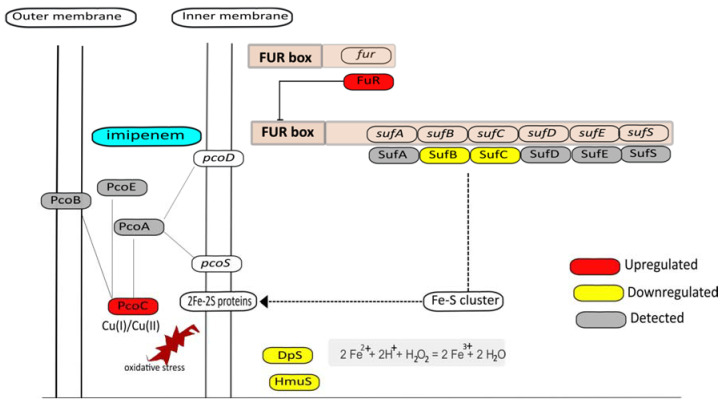
Graphical representation of the possible interrelationships of iron, copper, and Fur-regulated genes. Based on the quantitative proteome data, it is inferred that Fur represses the *sufABCDES* operon, leading to reduced synthesis of iron–sulfur cluster proteins in the membrane, thereby setting a limit on potential ROS formation by cellular respiration. Downregulated proteins are marked in yellow, including Dps and HmuS, where *hmuS* is probably repressed by Fur. Additionally, the periplasmic copper resistance protein PcoC was found to be upregulated, which might serve to protect remaining iron–sulfur clusters from copper-mediated destabilization and leakage of Fe^2+^ that could lead to Fenton chemistry.

Lastly, another response that can be related to the prevention of oxidative stress through reduced aerobic respiration in the presence of imipenem concerns the observed downregulation of SufB and SufC (Figure 7), which are involved in iron–sulfur cofactor synthesis [43,44]. This would lead to reduced electron transport in the membrane driven by NADH dehydrogenase, which contains an iron–sulfur cluster, like other proteins involved in respiration. In addition, reduced iron–sulfur cluster synthesis may also reduce the activity of the TCA cycle enzyme aconitase, leading to a lowered overall TCA cycle activity. Together, these observations imply that, combined with the aforementioned enhanced potential for vitamin K production, the bacteria in particular protect themselves against ROS produced at the membrane through respiration.

### 3.7. Metal Homeostasis

Kohanski et al. [6] have previously shown that many bactericidal drugs, including β-lactams, promote Fenton-mediated hydroxyl radical formation and that these events are mediated by the TCA cycle and transient depletion of NADH. In particular, iron may be leaking from iron–sulfur clusters, which would lead to the production of ROS by Fenton chemistry [44]. Hence, this could be a second reason why the biogenesis of iron–sulfur clusters with the aid of SufB and SufC is downregulated. A clue as to how this may be achieved by the bacteria is provided by the observed upregulation of the Fur repressor in imipenem-treated cells. Indeed, the *sufABCDES* operon of our *E. roggenkampii* study isolate is preceded by a typical *fur* box where the Fur repressor is predicted to bind. This enhanced activity of Fur is in fact confirmed by the observed downregulation of the ThrS and MaeB proteins, whose genes are also preceded by potential *fur* boxes. Likewise, the observed downregulation of the hemin transport protein HmuS is probably a consequence of the upregulation of Fur, as the *hmuS* gene is known to be Fur-regulated in other bacteria [45].

Interestingly, iron–sulfur clusters are highly sensitive to copper, which causes the release of Fe^2+^ and associated Fenton reactions [46,47,48]. Thus, another way to protect the cell against ROS formation would be the restriction of Cu. This could explain why the periplasmic copper resistance protein PcoC is upregulated in the presence of imipenem (Figure 7).

While the upregulation of Fur implies that the cells try to limit ROS production in the presence of imipenem, an important question that cannot be answered from our proteomics analysis is which signal exactly triggers the upregulation of Fur. Normally, this happens when insufficient amounts of iron are present in the cell. Therefore, we compared the metal content of the imipenem-exposed (at t = 30^+I^) and non-exposed control bacteria (at t = 30^−I^) by ICP-MS. Specifically, we could measure the abundance of two isotopes for iron (^54^Fe and ^57^Fe) but, unexpectedly, no significant changes in iron content were observed (Supplementary Table S3). At present, we do not know the reason for this finding, but it could be due to an imipenem-induced relocation of intracellular iron, where Fe^2+^ initially incorporated in other cellular compounds, such as iron–sulfur clusters, is sequestered to Fur. In turn, this would lead to the repression of Fur-controlled genes, like *sufABCDES*, leading to a downregulation of the synthesis of iron–sulfur clusters (Figure 7). In fact, this would be consistent with a scenario where imipenem triggers ROS production at the cytoplasmic membrane leading to the disintegration of iron–sulfur clusters. In this case, the ratio between Fur with or without bound iron would change, which is something we cannot detect in our MS analyses. Of note, the observed downregulation of the Fe^2+^-binding protein Dps would also be consistent with a relocation of Fe^2+^ to Fur. An intracellular relocation of iron would actually make sense in view of the fact that RPMI is an iron-restricted medium, similar to human plasma, in which bacteria need to carefully control their iron homeostasis [30]. However, we cannot presently exclude the possibility that the observed lack of effect of imipenem on the cellular iron content relates to the way in which the samples were processed for the ICP-MS analysis. On the other hand, we did observe a clear increase in the cellular copper (^63^Cu) content in the presence of imipenem (Supplementary Table S3), which is consistent with the upregulated level of the periplasmic copper binding protein PcoC, which protects the cell against copper stress [49].

### 3.8. Downregulation of Proteins Involved in Transcription and Translation

Lastly, among the 56 downregulated proteins in the presence of imipenem, various proteins involved in translation were identified. These include the leucine-responsive regulatory protein Lrp; the transcription termination/antitermination protein NusA; translation initiation factor InfB (i.e., a functional partner protein of NusA); the DNA-directed RNA polymerase subunit β RpoB; and the aminoacyl-tRNA synthases AlaS, AsnS, LeuS, LysS, and ThrS for the synthesis of L-Alanyl-tRNA, L-Asparaginyl tRNA, L-Leucyl-tRNA, L-Lysyl-tRNA, and L-Threonyl-tRNA, respectively. Consistent with the downregulation of the aminoacyl-tRNA synthases, the phosphorolytic 3’–5’ exoribonuclease Rph, involved in the tRNA 3’-end maturation, was also significantly downregulated. These observations suggest that the presence of imipenem may affect translation, although one has to bear in mind that, in our present experimental set up, this does not lead to growth inhibition, since a subinhibitory concentration of imipenem was used. Yet, the imipenem challenge was imposed on the cells over a time span of 30 min, which corresponds to about half the doubling time of our *E. roggenkampii* isolate in RPMI (i.e., 56 min). This would be consistent with the idea that the observed protein downregulation is, at least in part, due to reduced translational activity. In addition, it was previously shown for *E. coli* that severe oxidative stress impairs the editing activity of threonyl-tRNA synthetase, thereby causing protein mistranslation [50].

## 4. Conclusions

Altogether, our present observations show that exposure of a highly carbapenem-resistant *E. roggenkampii* isolate to subinhibitory amounts of imipenem triggers a set of very specific changes in the bacterial proteome. In particular, these appear to relate to the avoidance of oxidative stress by reducing the cellular capacity for glycolysis, TCA cycle, and respiration, thereby setting a limit to potentially detrimental ROS production at the cytoplasmic membrane. Consistent with this view, the cells limit the levels of proteins involved in iron–sulfur cluster formation, which, in turn, provides additional protection against ROS formation that may be caused by iron leakage from iron–sulfur clusters and associated Fenton chemistry. An additional advantage of reducing the capacity for glycolysis is that the cells will produce less intermediates for cell wall biogenesis, which will help to save cellular resources under conditions of impaired cell wall synthesis. Of note, while the observed ‘smart’ adaptations do not affect the growth of our *E. roggenkampii* study isolate, they do seem to make the bacterium fit for dealing with imipenem in its environment. This suggests that *E. roggenkampii* has the intrinsic potential to reach higher carbapenem resistance levels by metabolic adaptations than the here investigated isolate actually displays in our experimental setup. If so, the present study gives us an intriguing ‘sneak preview into the future of carbapenem resistance’ in a pathogen that is increasingly implicated in serious, difficult-to-treat invasive disease.

## Data Availability

The whole-genome sequence of the *E. roggenkampii* isolate 339389L is available from NCBI with the accession number CP026536. The mass spectrometry proteomics data have been deposited to the ProteomeXchange Consortium via the PRIDE partner repository [16] with the dataset identifier PXD013412 (Username: reviewer57181@ebi.ac.uk; Password: xEWohqZw).

## Supplementary Materials

The following are available online at https://www.mdpi.com/article/10.3390/antibiotics10050501/s1, Supplementary Figure S1: Voronoi treemaps presenting all proteins of *E. roggenkampii* ST232 as identified in our quantitative proteome analysis. Supplementary Figure S2: abundance of MIR17 in the investigated *E. roggenkampii* isolate. Supplementary Table S1: results from the SILAC analyses. Supplementary Table S2: results from the iBAQ analyses. Supplementary Table S3: results from the ICP-MS analyses. Supplementary Table S4: peptidoglycan synthesis-related proteins.

## Abbreviations

Abbreviations used are the following:

BSL-2	biosafety level 2
COG	clusters of orthologous groups
ICP-MS	inductively coupled plasma mass spectrometry
KEGG	Kyoto Encyclopedia of Genes and Genomes
LDS	lithium dodecyl sulphate
MS	mass spectrometry
OD_600_	optical density at 600 nm
PAGE	polyacrylamide gel electrophoresis
PBPs	penicillin binding proteins
PSSM	position specific scoring matrix
ROS	reactive oxygen species
RPMI	Roswell Park Memorial Institute 1640 medium
SILAC	stable isotope labeling of amino acids in cell culture
ST	sequence type
TCA	tricarboxylic acid
