# Supplementary material for: Proteomic Charting of Imipenem Adaptive Responses in a Highly Carbapenem Resistant Clinical Enterobacter roggenkampii Isolate
Source: Antibiotics (Basel). 2021 Apr 28;10(5):501. doi: 10.3390/antibiotics10050501 (PMC8145422; doi:10.3390/antibiotics10050501)
Supplement: Supplementary file 1 [file antibiotics-10-00501-s001.zip › Supplementary Figures S1 and S2.pdf]

Supplementary Figures S1 and S2

Proteomic charting of imipenem adaptive responses in a highly carbapenem resistant clinical *Enterobacter roggenkampii* isolate

Suruchi Nepal[1], Sandra Maaß[2], Stefano Grasso[1], Francis M. Cavallo[1], Jürgen Bartel[2], Dörte Becher[2], Erik Bathoorn[1], and Jan Maarten van Dijl[1#]

[1]University of Groningen, University Medical Center Groningen, Department of Medical Microbiology and Infection Prevention, Hanzeplein 1, P.O. Box 30001, 9700 RB Groningen, the Netherlands.

[2]Institute for Microbiology, University of Greifswald, Felix-Hausdorff-Str. 8, 17489 Greifswald, Germany.

[#]**Corresponding author**: [1]University of Groningen, University Medical Center Groningen, Department of Medical Microbiology and Infection Prevention, Hanzeplein 1, P.O. Box 30001, 9700 RB Groningen, the Netherlands. Phone: +31-50-3615187; E-mail: j.m.van.dijl01@umcg.nl.

Running title: Imipenem responses in drug resistant *E. cloacae*

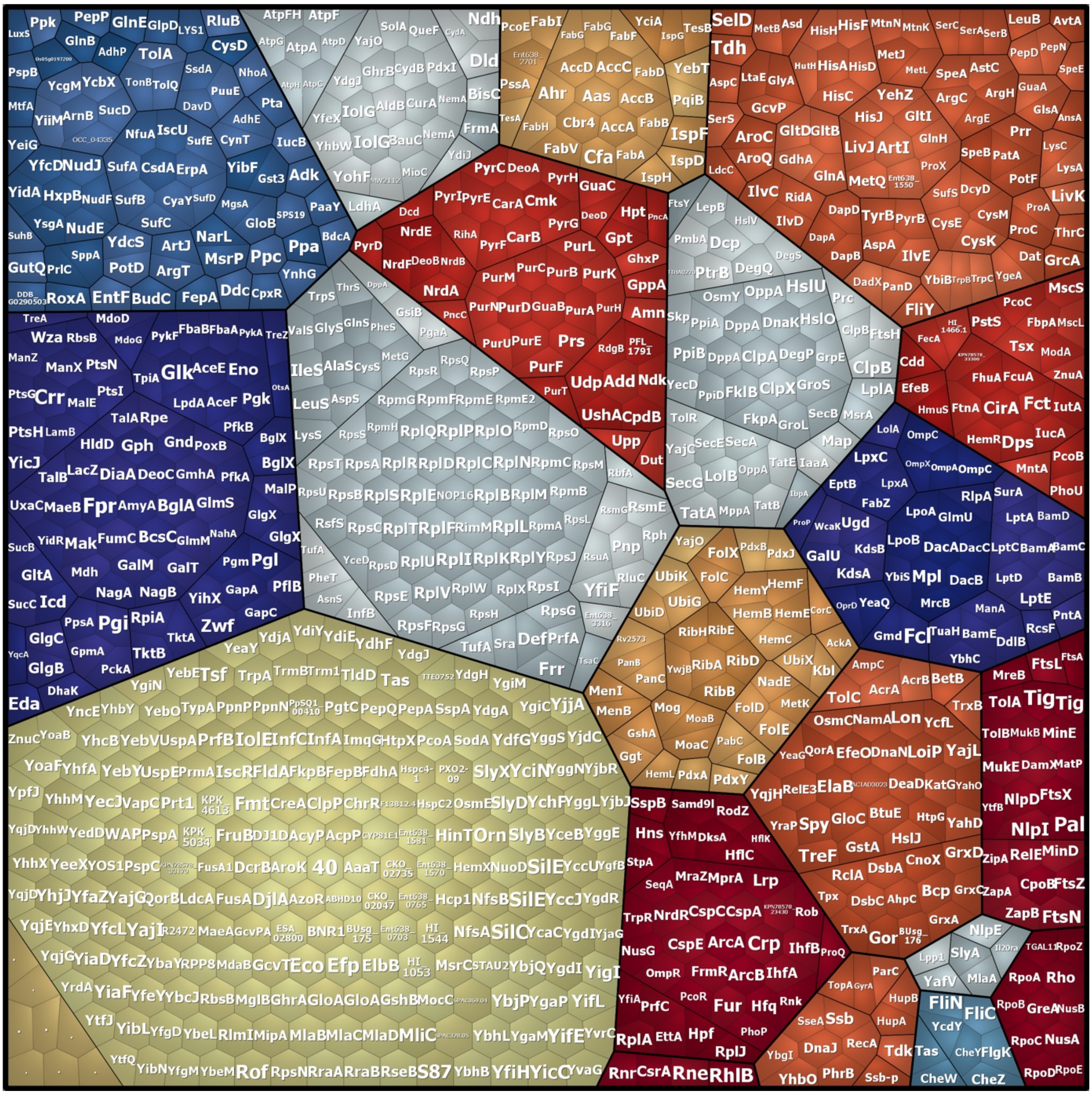

Supplementary Figure S1. Voronoi treemap presenting all proteins of *E. roggenkampii* ST232 as identified in our quantitative proteome analysis.

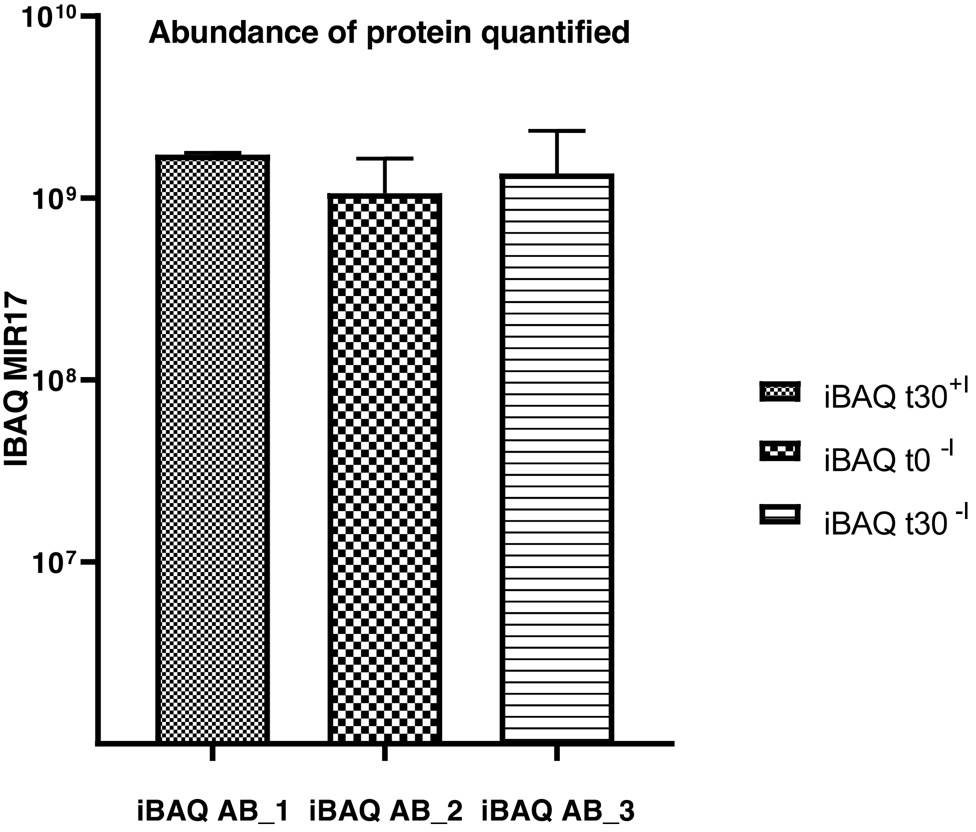

Supplementary Figure S2. Abundance of MIR17 in the investigated *E. roggenkampii* isolate.
